# The Diagnosis and Treatment of Alveolar Abscess

**Published:** 1911-09-15

**Authors:** Arthur D. Black

**Affiliations:** Chicago


### THE DIAGNOSIS AND TREATMENT OF ALVEOLAR ABSCESS.

BY ARTHUR D. BLACK, M.D., D.D.S., OF CHICAGO.

Read before the Tennessee State Dental Association, May 24th, 1911.

I wish to present the subject of the diagnosis and treatment of chronic alveolar abscess, not from the standpoint of the oral surgeon, but from the view point of a man who is in the general practice of dentistry. I wish to state a few simple facts and items in connection with the treatment, which I hope will be of some benefit to many of you in your practice.

We ought first in the treatment of chronic alveolar abscess to make a definite diagnosis of the conditions existing in a particular case. We must recognize that there are certain chronic alveolar abscesses which cannot be cured, and there is also another group of cases in which the chances of effecting a cure are good. In the beginning of the treatment of any case we ought to be able if possible to make a diagnosis which will enable us to put the case in one class or the other; and this should be done previous to undertaking any long series of treatments.

There was a time years ago when the dental profession could be seriously criticised for extracting many teeth which they ought not to have extracted; but I believe the time is here to-day when the pendulum has swung to the opposite extreme where we ought to extract many teeth that we are endeavoring to save, and thereby many times materially injure our patients' general physical health. I want to cite you one case that came to me in my own practice, a woman who had a history of tubercular infection and who had been supposedly suffering from tuberculosis some ten years, for a period of about three weeks she was having a temperature of a degree and a half to two degrees, and she had noticed that she was not feeling quite as well as usual. She had gone in much alarm to her physician as she was naturally considerably anxious over the probable return of her tuberculosis. She came about this time to me for an examination of her mouth, and I found there a chronic alveolar abscess, such as we all see frequently. I had an X-ray made of the case and it showed a little destruction of the bone about the root, and I extracted the tooth and the next day the patient was free from any fever temperature. So far as I know she had not had any discomfort with that tooth. We are inclined to carry a patient with a chronic alveolar abscess so long as teeth are not sore, so long as the patient is not uncomfortable on account of them. We know that a surgeon would not allow a patient to walk around the streets if he had an abscess discharging somewhere else about the body. Some measures would be taken to cure that patient, and I think we in the treatment of these chronic alveolar abscesses ought to cure them, even if that cure necessitates the extraction of the tooth. The matter of determining in the first place whether a case shall be put in the incurable or possibly curable class, the matter of a careful differential diagnosis becomes important. I think I do not misstate the case when I say it is too common a practice for us when we have a case presented with a chronic alveolar abscess, to look into the pulp canal of that tooth and possibly attempt the cure of the case by treatment of the pulp canal, without paying any attention whatever at that time to the condition that may be presented in the bone of the alveolar process and the tissues surrounding the apex of the root. Now it seems to me at the outset that our duty is plain that when a case presents itself in which there are signs of a discharge from an alveolar abscess that our duty should be to explore the sinus, without paying much attention to the pulp chamber and the root of the tooth. It is impossible for me to understand why so many members of the dental profession should wish to pass a probe through the sinus to make an investigation of the tissues there. I know that is a fact, for this reason, that in our school we have been trying for a good many years to teach our students to make the examination through the alveolar process the first thing, and while we have had a good many of them do this, there are a great many of them who do not. I know that because the percentage of cases that are sent into our clinic, cases in which men have never taken the time or care to make an investigation we have found a number of alveolar abscesses and I say where it is at the end of the root on an examination of the sinus, that is the one that should be made first and there are two instruments which should be used to examine all these cases; usually there are two, sometimes only one is necessary, that is if the sinus is one that is short and there is no question as to where that sinus leads, only one probe is necessary. But if there is some uncertainty about the direction of the sinus, the instrument to use is a small, blunt-ended silver probe, which is a probe with a soft blunt end, being passed into the opening in the sinus, and on account of the fact that it has a blunt end, and on account of the fact that it is soft it may follow the direction of the sinus without catching in the sides of the canal and without injury to the bone in the passage. As soon as that has been done, another probe should be used, a soft silver probe is almost useless for the purposes for which we want to use a probe in this case after we have located the other end of the sinus.

Some years ago Dr. Gilmore, of Chicago called attention to the use of a set of sharp steel probes for the purpose of making an exploration of this kind, a probe that any of you can make from an explorer, but about half an inch off the end of it, re-sharpen it and you will have an instrument that is the equal of any explorer sold by our dental supply companies. Pass this probe into the sinus until it comes in contact with the hard tissue, or with the end of the root bone and let that probe determine what is there. Now with such a probe as this you can easily make a differential diagnosis between the rough end of the root which is ordinarily absorbed, between caries of the bone, between the smooth end of the root of a tooth, and between a normal bone and the enamel of a tooth, which may be imbedded in the tissue. The blunt end passing over the surfaces or whatever you come in contact with, makes the diagnosis so easy and simple one almost feels foolish after using such a probe a few times.

In the case of the chronic alveolar abscess, this probe may be passed through the process and you usually come in contact with the end of the root. You can usually determine the course that should be pursued in the treatment of these cases. There is one thing that is of course impossible for us to know in connection with the treatment of these cases, and that is how much of the peri-dental membrane has been destroyed, that is how much of the membrane about the apex of the root has been destroyed. We all know from our experience that in cases of pyorrhea the peri-dental membrane is pulled away, detached from the bone and when it is broken and detached too long a time it forces the fibers to lose their vitality, and we cannot have a re-attachment of those fibers in exactly the same condition. And we have exactly the same condition at the end of the root. We know that when we extract a tooth it results in the absorption of the fibers of the peri-dental membrane which were attached to the tooth, also the absorption of the process to which those fibers were attached at the other end. We have exactly the same condition that takes place in pyorrhea, or chronic alveolar abscess. We have the tearing of the fibers from their attachment to the foramen that we have in the extraction of a tooth. We have all felt with the finger in passing over the alveolar process, we have felt the depressions there where the bones have been absorbed in cases of this type. Therefore, we cannot by any means hope to get a re-attachment of that tissue to the end of the root. Therefore the denuded portion must be removed because it is so connected as to be a continual cause of irritation to the tissues about it, and that to my mind should be the basis of our treatment. The first thing we should do should be to determine the amount of the end of the root that is nude as this practically passes through the tissues and comes in contact with them. And by pressure on the side of the root we can outline very closely the amount of the root that is denuded.

Diagnosis is helped by the use of the X-ray. All however, havn't access to good X-ray service, and unfortunately it is too expensive to send many patients to have radiographs made. The X-ray is not a necessity in the diagnosis of a large number of these cases, and we are sometimes deceived by the radiograph. It is often valuable in making a definite diagnosis, and there are some cases in which the X-ray is absolutely essential. I have some slides here of X-ray pictures of chronic alveolar abscess and in many of these cases the diagnosis was made almost as definitely with a probe previous to making the X-ray picture, as it was by the use of the X-ray.

Medication.—We know from experience and by the older custom of practitioners that ninety-five per cent phenol has been forced through the abscess tract as a treatment, and all kinds of antiseptics have been used for the same purpose, and these remedies are in common use by the profession to-day. We ought to follow our surgical friends in this regard, most of our surgeons are not using antiseptics in the treatment of wounds for the reason that it has been shown by many experiments that the use of any antiseptic will do more harm than good. If we use an antiseptic that is strong enough to destroy or even inhibit the growth of micro-organisms, it will also be strong enough to inhibit the healing functions of the tissue. In the practice of surgery and dressing wounds to-day, the method is to irrigate with sterile water to wash most of the germs, without doing any injury to the tissue. Some very interesting experiments have been made to prove the value of this plan. Without going too much into the detail in these matters I can refer to one series of experiments in which a number of pigs were taken and parallel incisions made in the back on each side of the spinal cord, through the skin. One of these wounds was treated by washing with water, and the others with first one and then another of the antiseptic solutions commonly used for this purpose, and the one that was washed with normal salt solution healed about two weeks ahead of the others.

Several years ago a cousin of mine suffered a gunshot wound through the forearm. My father and the surgeon who took care of him, were both very much interested in the study of the new ideas with respect to aseptic surgery. The operation was performed while the hands of the operator were subjected to a spray of carbolized water, and an attendant who was scattering a solution of carbolic acid around the room. In order to keep the arm in as antiseptic a condition as possible, they had made a tin tub so that the arm could be kept in a five per cent phenol solution, and the arm was left in that solution a day or two, and then they weakened the phenol solution, keeping this up altogether for a period of three weeks. During all that three weeks there was no infection of the arm, but there was no healing, the tissue of that arm had been so poisoned with the phenol that it was impossible for it to perform its normal function of granulation. After they took the arm out it was three weeks before any granulation was apparent, and it was not until they used dry dressings and less potent antiseptics that it healed.

One-half of one percent of phenol will so paralyze tissue that it will not make any attempt to recover an injury. We ought to take these things home to ourselves seriously and question whether or not we are doing the right thing with these sinuses when we subject them to such powerful destructive drugs. They are chronic abscesses, and in using strong drugs we must be doing that which really delays the healing of these wounds. If we use a normal salt solution we would find that these cases would be very much better than with any antiseptic solution. In our school during the past year we have for the treatment of all cases of pyorrhea, and all cases of chronic and other abscesses, and for all surgical wounds, used nothing but the physiological salt solution for irrigation, and there is no comparison between the recovery of these wounds and the wounds where we previously used antiseptics.

Now a word as to the treatment of the chronic abscess other than the medicinal treatment. In connection with the cure of any root, proper root filling must be made. In those cases in which we find by an examination there has been but little destruction of the tissue attached to the end of the root, we should then properly proceed with the treatment of the root canal with the exception of the end. But for the other group of cases in which we conclude that the treatment of the root canal will not alone cure the case, cases in which we have denudation of the end of the root, then I do not see how it is possible for us to hope for a cure while a denuded root remains in place. There are three different methods of treatment. The first, the simplest and in many cases the best is the extraction of the tooth, which I think all of us should more frequently do. The second is the amputation, specially of roots of certain teeth, the lingual roots of the upper molar teeth, and more especially of the upper first molar. Excision is the simplest of any of these operations and should be done in many cases, in which a long series of treatments would follow. The amputation of the lingual root of the upper first molar is more easily accomplished than it is in the second, for the reason that the bifurcation is closer up to the front than it is in the second molar. After the diagnosis has been made, take a pair of right and left curved explorers, and introduce one of these in from the distal side, and carry it around by the lingual root so as to get in close to the crown of the tooth, and then introduce the other one from the mesial side of the tooth and do the same thing that you did on the other side, and you get up close to the crown and to the bifurcation, you will need no local anesthetic. I have amputated many such roots without telling the patient what I was going to do until after it is done and the end of the root was removed. Take a bi-bevel drill directly through the root linguo-buccally, then take a fissure burr and finish the amputation. After which use a large spoon excavator, and lift the root out after it has been separated from the crown. It takes but a minute to put a gutta-percha filling in the canal in the crown portion, and finish out the filling with non-cohesive gold and you will have left a nice gold filling in the exposed opening of the root canal. For lower molars where one root was in such a condition that it needed to be removed, and the other was in good condition, I usually removed the portion of the crown that corresponded to that root, for example if it was the distal root of the lower first molar, I would remove the distal part of the crown. Also I believe you can usually get a better result in doing that than to attempt to make the amputation from the lingual side of the tooth.

Then for the third operation, I wish to mention the resection of some of the single rooted teeth. These teeth are difficult to amputate and we have failures because we have to cut through so much bone to get to them, but for the principal six front teeth this operation is very satisfactory. I have had one cut off in my own mouth and the tooth has done very good service since, and I doubt if any one here could find it. I have had a few cases in which the roots were resected in this way as long as nine or ten years ago. The operation of amputation and resecting the end of the root is not much different from the amputation of the lingual root of the upper molar. Make an incision through the soft tissue exposing the root, which is usually already exposed, the bone having been destroyed, and then cut it off with a fissure burr making the end somewhat round. In some of these cases there may be a complication of caries of the bone, but I think we would be safe in saying that in more than ninety per cent of these cases where the root end alone is removed the bone will take care of itself, and we will get a healing. The operation of resecting the end of the root should be confined pretty closely to the upper six front teeth. Of course where any one of these operations is performed it should be done so as to leave enough of the peri-dental membrane so that it is not loose. If the tooth is much loose I believe we had better extract in the beginning.

DISCUSSION.

Dr. D. M. Cattell.—It is almost impossible that such a paper could be discussed other than to give it commendation. I want to emphasize the importance of right diagnosis. I notice in general practice that there is a great discrepancy in methods of diagnosis in practice. There is no such examination of the case as a surgeon would make. We too often treat by routine or habit. We do not examine carefully to know conditions much less to anticipate results of treatment, in fact there is no study of the case. We need to attack these cases just as a real surgeon would. Our diplomas all say that we are dental surgeons, but so many of us after all are not. We do not examine our cases as a scientifically educated man should do. I am thankful to know that some of us do this. Few dental schools teach thorough diagnosis; there are a few that have such chairs and special stress is put upon diagnosis and prognosis, so that when their young men go out into the practice they have been trained to take proper precautions from the start. They should be able to follow the course of an abscess, so they can see it step by step in their mind's eye, so to speak, what it might be or what a sky-graphic view of it might represent. Usually all they do in a chronic abscess is to drill into the pulp chamber and begin the treatment of the root canal and later hope to treat the abscess through the alveolar sinus. I shall try Dr. Black's suggestion of making the first examination through the sinus in cases of chronic abscess with a sinus. My plan had always been to examine everything through the canal first and see if there was any prospect of treatment and recovery through the canal, and in case I make little progress I then explore through the sinus. I judge from his experience and a large clinical observation it has been proved to him and his confreres that this method is best. In regard to extraction of teeth that can't be cured, the essayist has recommended a procedure that at first sounded somewhat strange coming from so conservative a man. I have no doubt we try to save many teeth that we had better remove at once. We should not undertake the saving of teeth that there is little prospect of our ever making useful or healthy as they may prove a focus of infection. When we are satisfied that a tooth can not be made healthy, whether it is needed or not the only thing to do is to extract it. I have believed that a slight issue of pus may exist in the mouth for years and do no damage. The use of that tooth even in that condition may be useful to the patient, while if the tooth is extracted its usefulness would be gone. I have not been so radical in my practice and in my belief as the essayist. There is a point that I believe that many practitioners often recognize and the essayist asserted that many dentists believe that there would be a renewal of the peri-dental membrane after there has been extensive destruction of it by disease. I do not believe there will be any regeneration of the peri-dental membrane after it has all been wasted away. I have no doubt however that there may be in favorable circumstances considerable regeneration if there be some healthy tissue to start with.

I am glad the essayist brought out the point of the excessive use of drugs. I remember when I came into the profession we had to have drugs, oil of cloves, creosote, carbolic acid and iodine, and some few had a little more, but those were the main standbys. As time went on we have added another thing, and another until now we have to have a small drugstore. We used to have in Chicago a dentist who used to delight in adding to our list of drugs, he was working, searching for something better than what we had, and finally it was given out that a man couldn't practice dentistry unless he had at least twenty-two different drugs or remedies in his office. To-day we have swung off a good deal and most of us get along with a half dozen drugs and these mostly proprietary remedies, but we also use more water and antiseptic solutions for irrigation. I remember when we used as a douche a five per cent solution of phenol, and now we are finding the normal salt solution better. It has been talked of a little in our profession, but the medical and surgical professions are making large use of normal salt solution. If the normal salt solution is as good as the stronger drugs we have used, it certainly should be preferred. I have believed in root amputation for many years successfully. Many dentists seem to be afraid of the knife, or the blood or they are afraid of cutting, they are afraid of doing anything unless they do it directly to the tooth itself; but they are not afraid to clean out a cavity or dig through the pulp chamber and canal. I have seen so many cases of the amputation of roots that have been under the influence of pus, chronic abscess, etc., that have resulted satisfactorily, that it seems to me such a simple and safe operation compared to many others that we do. I remember one case where there was an abscess of long standing, and not only the root of the lateral incisor was involved but one of the cuspids. The patient had been under treatment for a long time, pus was flowing just as readily after many months of treatment as before. The incisor root was cut down almost to the gingival line, so near the gingival line that I felt that the crown must soon give way. The central incisor root was amputated one-fourth, the cuspid at least one-third. The case was cured and I saw the patient last January, and that lateral incisor is still doing service, and that was ten years after the operation.

I feel that I have not said very much, but I did want to express my commendation of Dr. Black's methods. I want you to know that Dr. Black was formerly one of my students.

Dr. Leonard: I rise to commend the essay as a whole, I think it is one that is worth a great deal to every member who has heard it. There are one or two points, one point particularly, that I can scarcely reconcile with my own clinical experience. In reference to the use of drugs in the treatment of chronic abscesses, I take it that in most cases there is a fistulous opening or a fistulous tract leading to the gum surface. In such cases where the appearance would indicate a very great destruction of tissue, and where there is not very much inflammatory trouble, and where there is only a small discharge of pus through the opening in the gum, or by the side of the root of the tooth, it has always been my practice to open the canal and cleanse it as well as I can and force through it some agent to destroy the infection, and at the same time destroy this suppurating membrane. I have used for this purpose carbolic acid, not dilute, but full strength, with the belief that this simply destroys this suppurating membrane just as we would do it mechanically, and as this is a liquid it will follow the tract much better than we can with a probe or than we can with an instrument and destroy it mechanically. This has been the treatment that I have applied almost uniformly throughout my professional experience and with almost uniformly good results. In cases where there is a denuded surface, or where there is a deposit of calcareous matter, or where there is cancellous bone, it will not cure such cases. I think the amputation of the root in such cases is the most logical treatment, and it is certainly one that should bring satisfactory results. The treatment I mentioned is very simple, and it is very easy to make this test. If it should not penetrate the sinus we should feel pretty certain that there is some foreign materials at the end of the root; either the root is denuded, or there is some deposit of calcareous matter, or possibly some root filling has forced through. In case I can not get positive results from this treatment of this fistulous tract then I should certainly adopt Dr. Black's plan and make an exploration with a probe. We ought to be more careful in diagnosing our cases to find out before beginning just what the conditions are and then we can treat from the most intelligent standpoint. I would like to ask Dr. Black in the case next to the last he has shown, if there was any history of syphilitic infection in that case.

Dr. Black: Not that we found. It was a clinic case and the patient did not have any syphilitic infection. I think it was a case where the pus got above the attachment of the muscles and the pressure carried it on up to where it came out.

Mr. President, I believe that Dr. Leonard's discussion is as nearly the condition I am fighting as anything that could have been presented. Dr. Leonard says that he cleans out the root canal and forces some drug through the canal and through the sinus and then if it does not get well he gets an explorer or probe to find out what is the matter. Now why shouldn't you probe first? It does not hurt the patient to carry a probe up through the gum, and why can't you find out first what the condition is before doing anything at all? In many cases if you will carry the probe in first and make a thorough examination, you will find that the tooth ought to be extracted, and if so, you have been wasting a lot of time going through those things. That is the point I wish to make now. He says he forces ninety-five per cent phenol through the tract, and in many cases they get well, and if they don't get well then he finds out there is something else the matter at the end of the root. I would like for him to study the question, take the next ten or fifteen cases and force normal salt solution through instead of phenol, and see if they don't get well quicker than they did before. Your phenol has only been good where there has been no trouble outside of the root or where there has not been much trouble. You have removed from the pulp chamber the source of the trouble and then you have washed out the tract with the phenol. I believe if you didn't wash it out with anything at all it would get well, but the use of anything to wash that out will remove the bacteria from that sinus, and that is the most important thing to do, and I believe you only delay the healing a little by cauterizing it with the phenol solution. If the source of infection is removed then the sinus will heal up. I believe you can convince yourself of that by a very little trouble; if you will take, say the next ten or fifteen cases you find and use normal salt solution only instead of phenol.

Dr. Leonard: How about Beck's paste?

Dr. Black: In the first place I would say that Beck's paste was brought to the medical profession and then the dental profession because some of it was used in some tubercular sinuses and the physician was surprised to find that some of these got well, and therefore he went on injecting everything that came into his office. I have watched Beck's paste pretty closely in dentistry and I know that there are quite a few men who have tried to use and have studied it and every one of those men without any exception have stopped the use of it. I am not saying that there is no merit in that paste, I don't know, but I do know from the experience of several men whom I have been associated with, who are pretty careful observers, that they started out with a great deal of enthusiasm for it, and now they are using it only occasionally in case where the pain is limited in the jaw so that there is no bone exposed. Possibly I am not giving Beck's paste a fair deal in what I say, but that has been my experience.

Dr. Johnson: What do you use for root filling?

Dr. Black: Gutta-percha.

Dr. Leonard: Can you tell us something about cystic conditions at the end of a root resulting from an abscess?

Dr. Black: I want to answer one other question first, please. My brother is a surgeon and he had an operation for a woman for appendicitis, and he was going to use an injection of a half pint of normal salt solution below the right breast; the nurse in making up the solution attempted to inject a saturated salt solution, which is about a twenty-five per cent solution, while the normal salt solution is half of one per cent, making this solution fifty times as strong as it should have been. There was a slough as large as my hand when I saw the case, of all the tissues, down to the ribs, you could lay your hand in that wound. The slough had extended up into the costal region and a great part of the tissue has been removed, so that the woman is very lame. That was an absolutely dry slough, no pus forming whatever, but the woman is going to recover. We have no stronger poison hardly than salt, therefore, salt solution should be used very carefully. The nicest way is to make a saturated solution and that should be filtered, no need to bother about boiling your water if you are going to use a saturated solution, if you are going to make a saturated solution you need not be afraid of any infection. This gives a very beautiful clear solution, which makes a solution of about twenty-five per cent. You take out about a teaspoonful to a glass of water to make your solution. It is not absolutely necessary to have an exact normal salt solution for our work, if you are below one per cent you are on the safe side.

Dr. King spoke of those cases where there is a slow discharge but no discomfort and the tooth is giving good service. We know of many cases of this kind where there seem to be no ill effects from it, but I do not know how we are going to know whether the patient is suffering or not, whether the patient's general physical condition is being injured by such a condition or not.

I want to tell you of a case of a woman who lives in Winnepeg, who had been suffering for nearly two years with very severe headache. She was in a hospital three months, had two or three operations performed with the expectation that they would relieve these headaches. She got no relief from it whatever. Accidentally she went to a young dentist who was a graduate of our school, to have something done to a lower tooth, and in looking at her mouth he saw signs of an upper left incisor being involved, and upon asking her about it, she said, "Oh yes, I know that has been there for a good many years, but it does not bother me." He passed a probe into it and found the pus cavity. This young man asked me to see this case, which I did, and I told her that I thought that was the cause of her headache, and we extracted the tooth. That was last December and she has not had a headache since. Now that is just an example of one of the cases where there were a half dozen who had seen that patient and examined that tooth, talked to her about the sinus, and had passed it over as we are accustomed to do many of ours, yet that woman was in a very serious nervous condition on account of that tooth. I really believe that we have gone too far in our attempts to save teeth with chronic abscesses. I do not know where to draw the line. It may be that an attempt to save the tooth will be justified in some cases, I believe we have injured the lives of some of our patients by trying to save these teeth. Our main work has been to save all our patients teeth and keep their mouths in good condition to chewing. On the other hand I suspect that the profession as a whole in an earnest endeavor to save all the teeth has jeopardized many patients' physical welfare.

I said what I did to Dr. Cattell this morning regarding the impossibility of the renewing of the peri-dental membrane, because I have heard twice during the last year that there is a man who makes a specialty of the treatment of pyorrhea, and he said in open society meeting that he cured cases of pyorrhea. When they can secure the reattachment of the tissues to the root of the tooth, I think it is worth while, but it is a physiological impossibility for that tissue to be re-attached after the tissues have lost their vitality. Now there are fibers that surround the root of the tooth, and if these fibers themselves have been destroyed, how is it possible for them to be rebuilt? It is absolutely impossible in my opinion.

In regard to the prognosis of the resected ends of the roots, we should be a little careful to tell our patients that the result of such an operation is not exactly positive, it may last a year or a number of years, it may last longer than a year. We should tell our patients that here is a case that we must extract this tooth now, or we must do this operation, and if the operation will save the tooth for even a few years, it is worth while to do it.

We still have a few men who are doing a good deal of replanting of teeth; as a general proposition I do not believe in the replantation of teeth, because a very great majority of these teeth are lost by absorption if they are replanted or transferred.

